# Quantifying the Advantage of Vector over Scalar Magnetic Sensor Networks for Undersea Surveillance

**DOI:** 10.3390/s26041290

**Published:** 2026-02-16

**Authors:** Wenchao Li, Xuezhi Wang, Qiang Sun, Allison N. Kealy, Andrew D. Greentree

**Affiliations:** 1 School of Science, RMIT University, Melbourne, VIC 3001, Australiaxuezhi.wang@rmit.edu.au (X.W.); qiang.sun@rmit.edu.au (Q.S.); 2 Innovative Planet Research Institute, Swinburne University of Technology, Melbourne, VIC 3122, Australia; akealy@swin.edu.au

**Keywords:** magnetic sensing, target tracking, sensor networks, unscented Kalman filter

## Abstract

Magnetic monitoring of maritime environments is an important problem for monitoring and optimising shipping, as well as national security. New developments in compact, fibre-coupled quantum magnetometers have led to the opportunity to critically evaluate how best to create such a sensor network. Here we explore various magnetic sensor network architectures for target identification. Our modelling compares networks of scalar vs. vector magnetometers. We implement an unscented Kalman filter approach to perform target tracking, and we find that vector networks provide a significant improvement in target tracking, specifically tracking accuracy and resilience compared with scalar networks.

## 1. Introduction

Monitoring of maritime traffic is important for a range of applications, including managing congestion, identification of threats, and national security. As most maritime platforms have some magnetic signature, magnetometry is an important sensor modality for such surveillance [1,2].

Magnetometers for maritime surveillance can either be mobile platforms, for example aerial drones [3], uncrewed undersea drones [4,5,6], or towed arrays [7]. Alternatively, for specific regions of interest, magnetic trip lines [8] or arrays [9] can be considered. A magnetic trip line is a line or network of magnetometers that might be placed on the sea bed. The magnetic signals detected are then correlated to determine when an object passes over the sensors. This is the focus of the current work. Because the magnetic field from metallic objects decreases as the cube of the distance, magnetic sensor networks are most useful for monitoring in relatively shallow water.

Deployment of traditional magnetometers for long-term undersea monitoring is relatively costly due to the size and power requirements of magnetometers with sufficient sensitivity. Quantum approaches provide a new context to explore undersea magnetometry due to their reduced size weight and power for the same sensitivity as traditional classical magnetometers. In particular, optically pumped magnetometers [10] provide outstanding scalar magnetometry, with sensitivity reaching down to tens of femtotesla per square root Herz [11,12]. New generations of diamond magnetometers also provide outstanding sensitivity including vector sensing [6,13], with record sensitivity of around $500\;\mathrm{fT}/\sqrt{\mathrm{Hz}}$ [14,15]. Diamond in fibre approaches promise robust housing and low size, weight and power (SWaP) [16,17,18,19,20]. Though the sensitivity is not as high as conventional diamond magnetomers, diamond in fibre can still achieve 30 pT/$\sqrt{\mathrm{Hz}}$ [21]. These levels of sensitivity should be compared with the expected noise floors in marine environments, which are typically around the hundreds of pT level [22,23], depending on the conditions. Such noise can also be mitigated by emerging de-noising techniques [24], although we will not consider such details here.

Here we show a comparison of magnetometer sensing networks with a particular emphasis on sensitivity at current or near-future sensitivity for quantum sensors. We compare the efficiency of target tracking for single-path and periodic targets, quantifying the improvement through use of vector vs. scalar sensors. We find that vector sensors significantly outperform scalar sensors, by more than a factor of three. These results show that a sparser vector network can outperform a more dense scalar network, which has implications for the robustness and cost of implementing and operating such magnetometer networks for persistent monitoring applications.

This manuscript is organised as follows: we first introduce our measurement and system models; we then describe our centralised unscented Kalman filter; lastly we introduce two measurement scenarios.

## 2. Problem Formulation

**Figure 1:**
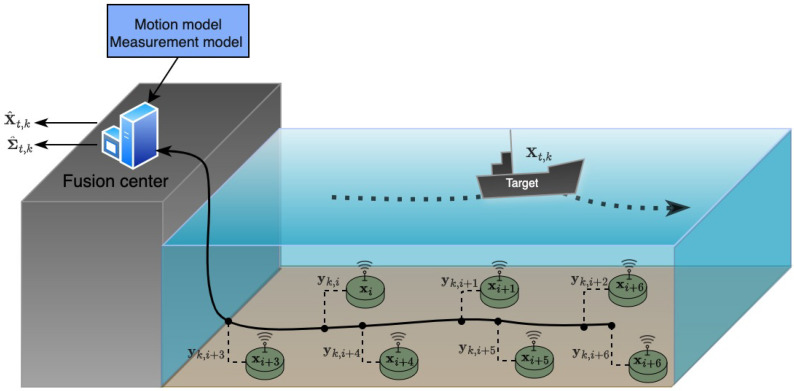
Estimating the target’s true location $X_{t,k}$ at time *k* via centralized fusion and a sensor network composed of *n* sensors. These sensors are located at $x_{i}$ and can provide magnetic scalar measurement or magnetic field measurement to the fusion center for tracking a target.

In general, targets of interest for tracking will have a non-trivial magnetic signature. Nevertheless, when the distance between target and sensor is three times longer than the size of the target, we can approximate the target as a single magnetic dipole [25], i.e., far-field assumption. For example, when the depth of sensor is 24 m, this assumption holds when the size of the ship is less than or approximately equal to 8 m. Small or medium/standard Autonomous Surface Vehicles (ASVs), such as C-Enduro [26], DriX [26] and SailDrone [27], fall into this category. In the near-field case, where the distance is closer or the target size larger, the modeling error of the single dipole approximation could be significant [28]. In such cases, higher-order multipole models should be considered in the measurement model of the filter. Such treatments will not change the overall conclusions of our work, although they will alter the numerical results. A schematic of our system is shown in Figure 1.

At time k=1,2,3,⋯, the location of the target dipole is $x_{t,k}={\left[x_{t,k},y_{t,k},z_{t,k}\right]}^{T}$, where the subscript *t* in $x_{t,k}$ indicates the target. The motion of the target is described by the state

(1) $$\begin{array}{c} X_{t,k}={\left[x_{t,k}^{T},{\dot{x}}_{t,k}^{T},{\ddot{x}}_{t,k}^{T}\right]}^{T} \end{array}$$

where ${\dot{x}}_{t,k}$ and ${\ddot{x}}_{t,k}$ are the target’s true velocity vector and acceleration vector respectively.

The magnetic field from the target dipole as measured at the *i*-th static sensor at location $x_{i}={\left[x_{i},y_{i},z_{i}\right]}^{T}$, i=1,2,⋯,n, is [29,30]

(2) $$\begin{array}{cc} B({\bar{x}}_{i,k})= & \frac{\mu_{0}}{4\pi }\left(\frac{3\left(M\cdot {\bar{x}}_{i,k}\right){\bar{x}}_{i,k}}{r_{i,k}^{5}}-\frac{M}{r_{i,k}^{3}}\right)\\ = & \frac{\mu_{0}}{4\pi r_{i,k}^{5}}\left(3{\bar{x}}_{i,k}{\bar{x}}_{i,k}^{T}-r_{i,k}^{2}I_{3}\right)M \end{array}$$

where ${\bar{x}}_{i,k}={\left[{\bar{x}}_{i,k},\;{\bar{y}}_{i,k},\;{\bar{z}}_{i,k}\right]}^{T}=x_{i}-x_{t,k}$ is the vector between the ith sensor and the target at time *k*, $r_{i,k}=\left|\right|{\bar{x}}_{i,k}{\left|\right|}_{2}$ is the sensor–target distance at time *k*, and $I_{3}$ is the 3-dimensional identity matrix. $M={\left[M_{x},M_{y},M_{z}\right]}^{T}$ is the magnetic moment of the target dipole and $\mu_{0}$ is the permeability of the medium, assumed to be sea water.

We treat two different measurement cases as follows.

- Scalar measurement:
  The case of scalar measurements corresponds to sensing via scalar magnetometers, for example optically pumped magnetometers [31]. In this case, at time step *k*, the *i*-th sensor measures the norm of $B({\bar{x}}_{i,k})$, i.e., $∥B\left({\bar{x}}_{i,k}\right){∥}_{2}$. The measurement model becomes
  (3) $$\begin{array}{c} y_{i,k}={∥B\left({\bar{x}}_{i,k}\right)∥}_{2}+\omega_{i,k} \end{array}$$
  where $\omega_{i,k}∼N\left(0,\sigma^{2}\right)$ is the noise term with zero mean and variance $\sigma^{2}$.
- Vector measurement:
  The vector case corresponds to an array of vector magnetometers, such as are realised by nitrogen-vacancy diamond magnetometers [32]. The vector measurement model for sensor *i* at time step *k* is
  (4) $$\begin{array}{c} y_{i,k}=B\left({\bar{x}}_{i,k}\right)+\omega_{i,k} \end{array}$$
  where $\omega_{i,k}∼N\left({\left[0,0,0\right]}^{T},\Sigma \right)$ is the noise term treated as Gaussian distributed with with zero mean, and variance matrix $\Sigma =\sigma^{2}I_{3}$, where σ is the noise variance.

**Remark** **1.** *As scalar and vector sensors typically use different technologies, there is no guarantee that the noise levels will be comparable. However, for simplicity in our analysis and to provide fair comparisons between implementations, we assume that the variance matrix *Σ* in *(4)* is a diagonal matrix with identical diagonal elements that is equal to the noise variance in the scalar case *(3)*, i.e., $\sigma^{2}$.*

*On the other hand, the noise characteristics of the sensors may be significantly different for each axis, and may vary between sensors and according to their local environment. Therefore calibration should be performed before and/or during deployment for each sensor to identify the noise characteristic. The associated calibration methods can be found in [33,34].*

The moment M should be estimated as it is a crucial parameter in the filter. In practice, it can be estimated using the measurements of the sensor network via the Least Squared (LS) estimator, i.e.,

(5) $$\begin{array}{c} \left\{\hat{M},{\hat{x}}_{t}\right\}=\arg\min_{M\in R^{3},x_{t}\in R^{3}}\sum_{i=1}^{n}{∥y_{i}-B\left(x_{t},M\right)∥}_{2}^{2} \end{array}$$

where ${∥\cdot ∥}_{2}$ is 2-norm. This LS problem can be solved via simulated annealing algorithm given in [35] reliably.

**Figure 2:**
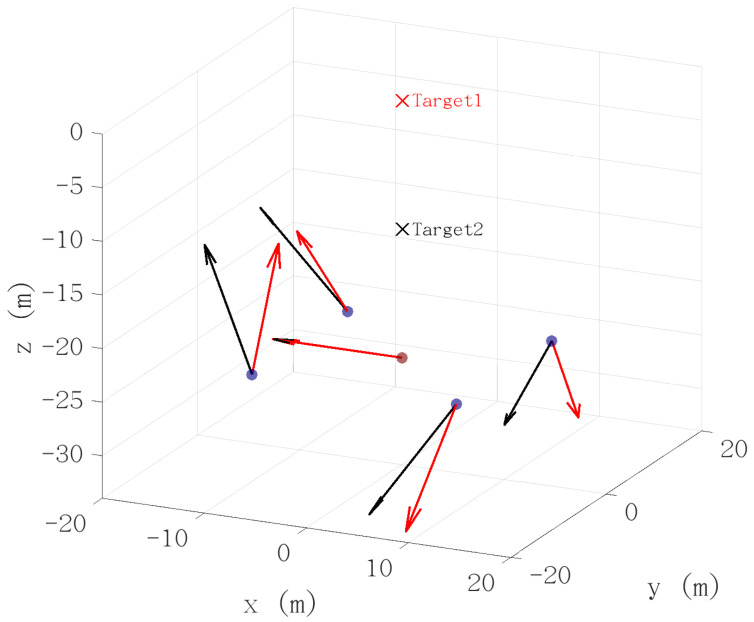
Difference in signals in sensor array due to two different magnetic targets. The signals at each node are indicated by the arrows—red for target 1, black for target 2. In this case the target positions and magnetic moments are such that the signals at the central node (brown dot) are identical for each target. As can be seen, the signals at the other sensor locations (blue dots) show different (vector) signals, shown by the non-overlapping arrows. These results demonstrate the ability for a sensor network to decouple magnetic moment from target location.

A single sensor is unable to decouple target position from magnetic moment M, and hence will always be limited when tracking unknown targets. However a sensor network is capable of resolving this ambiguity. For example, consider two dipoles located at different positions: target 1 at ${\left[0,0,0\right]}^{T}$ and target 2 at ${\left[0,0,-12\right]}^{T}$ m. The magnetic moment of target 1 is $M_{1}={\left[1,0,0\right]}^{T}$ A·m^2^, while that of target 2 is $M_{2}={\left[1/8,0,0\right]}^{T}$ A·m^2^. Suppose that five vector sensors are deployed at [0,0,−24] m (center sensor), [10,10,−24] m, [10,−10,−24] m, [−10,10,−24] m, and [−10,−10,−24] m. In this configuration, the magnetic field measurements produced by the two targets at the center sensor are identical, namely ${\left[-72.34,0,0\right]}^{T}$ μT. Nevertheless, the two targets remain distinguishable, and their magnetic moments are observable when measurements from the peripheral sensors are incorporated. This scenario is illustrated in Figure 2. It can be observed that the center sensor, shown in brown, yields identical measurements for both targets, as indicated by overlapping quiver arrows, whereas the peripheral sensors, shown in purple, provide distinct measurements that enable ambiguity resolution and moment estimation.

Nonetheless, in this manuscript, we are aiming to demonstrate the efficiency of vector sensors via comparing with scalar sensors. The inaccuracy of M is out of the scope of this paper and will be included in the future research.

Equation (4) assumes that the underlying three-axis magnetometer is placed exactly aligned with the coordinates of the Earth’s magnetic field. In practice, alignment error, which is often termed the sensor attitude error, cannot be avoided in sensor placement. We therefore consider the effects of small and uncalibrated sensor alignment errors. We treat these errors by determining the equivalent measurement noise induced by the misalignment, and adding this to the overall measurement error budget.

Let Δθ be the Euler angles between the local magnetic field frame of the earth and the magnetometer axes, which we call the sensor attitude error, SAE. C(Δθ) is the direct cosine matrix associated with that SAE. For a sensor with non-zero attitude error, the measurement equation at position $x_{i,k}$ is given by

(6) $$\begin{array}{c} y_{i,k}^{m}=C\left(\Delta \theta \right)B\left({\bar{x}}_{i,k}\right)+\omega_{i,k}. \end{array}$$

Compared with the ideal measurement calculated using Equation (4), the measurement difference is

(7) $$\Delta y=y_{i,k}-y_{i,k}^{m}.$$

**Figure 3:**
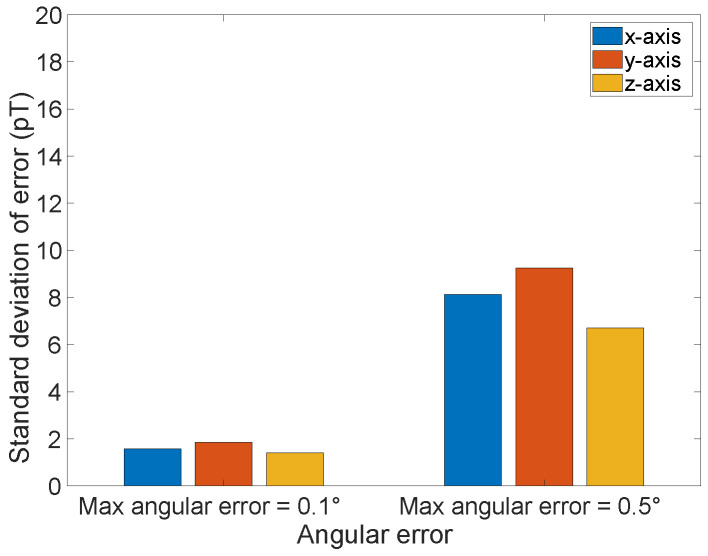
Equivalent three-axis error of the vector magnetometer with random angular errors applied to three rotation axes, where the angular errors are drawn from 3-dimensional uniform distribution with max angular error $0.1^{\circ }$ and $0.5^{\circ }$.

The measurement difference becomes an effective unknown term, due to the statistical variation in the SAE, and therefore is incorporated into the equivalent sensor noise. Figure 3 shows the results of 1000 Monte-Carlo simulations to generate the additional sensor error due to the SAE as a function of the magnitude of the angular error for two different error magnitudes, $0.1^{\circ }$ and $0.5^{\circ }$, calculated using (7). Each case took the average of 10,000 $x_{i}$ that were randomly drawn in the area of [−400,400]×[−400,400] m^2^. As is shown in the figure, a $0.1^{\circ }$ error corresponds to an error around 2 pT in each axis, and $0.5^{\circ }$ corresponds to an error around 8 pT. Such errors are small compared to the sensor noises we have assumed, and the expected undersea environmental noise. Nevertheless, such errors may need to be considered in the overall error budget; however, in what follows, we will not consider any additional effects due to SAE with the assumption that the entire error budget is due to intrinsic sensor noise.

**Figure 4:**
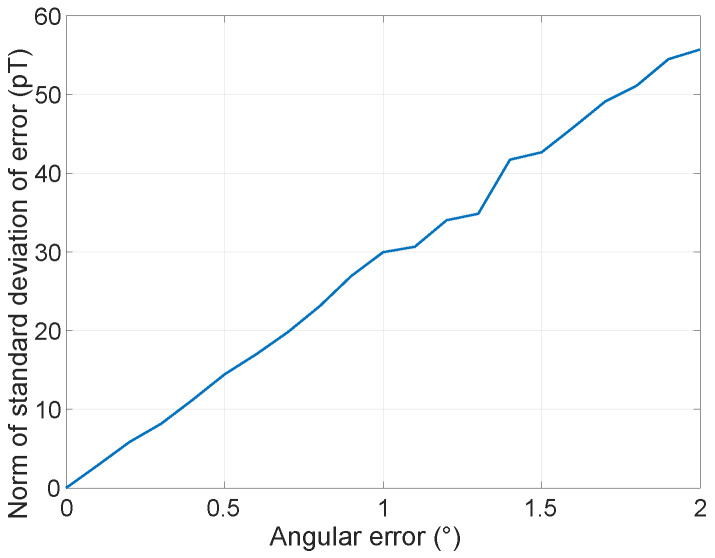
The norm of standard deviation of equivalent magnetic field errors as a function of SAE. In this low angular error limit, the equivalent magnetic field error grows linearly with SAE.

Additionally, the norms of standard deviation of the errors are plotted against SAE ranging from $0^{\circ }$ to $2^{\circ }$ in Figure 4. It can be seen that the error increases with the SAE and should be considered when it is large.

Considering the state of a target at time *k*, $X_{t,k}$ defined in (1), the state–space system with scalar measurement or vector measurement can be modeled as

(8) $$\begin{array}{c} X_{t,k}=f\left(X_{t,k-1},u_{k}\right) \end{array}$$

(9) $$\begin{array}{c} y_{i,k}={∥B\left({\bar{x}}_{i,k}\right)∥}_{2}+w_{i,k}\;\;\mathrm{or}\;\;y_{i,k}=B\left({\bar{x}}_{i,k}\right)+\omega_{i,k} \end{array}$$

where (8) describes the motion of the vehicle, (9) is the measurement model, $u_{k}$ is the process noise which is assumed to be Gaussian distributed with zero mean and covariance $Q_{k}$ and ${\bar{x}}_{i,k}=HX_{t,k}-s_{i}=x_{t,k}-s_{i}$ where H is given by

(10) $$\begin{array}{c} H=\left(\begin{array}{c} I_{3}\;\left|\;0_{3}\;\right|\;0_{3} \end{array}\right), \end{array}$$

and is used to extract the target position from the full target state, where $0_{3}$ is the 3-dimensional zero matrix.

The target tracking problem with the model (8) and (9) is to find the posterior density of target state over time based on the collection of magnetic field measurements from the sensor network, i.e., $p(X_{t,k}|y_{i,j},i=1\cdots ,n\;\mathrm{and}\;j=1,\cdots ,k)$. In the following section, we solve this problem using a Bayesian centralized data fusion, where an unscented Kalman filter (UKF) is used for nonlinear state estimation of the target. The estimators are established for both the scalar and vector measurement models.

## 3. Centralized Unscented Kalman Filter

Our system considers a sensor array in a network. To perform target tracking, we assume that all sensors report their measurements to a common center for processing and tracking, i.e., centralised tracking. Since the measurement is highly nonlinear as seen in (9), a nonlinear filter, such as extended Kalman filter (EKF), unscented Kalman filter (UKF), or particle filter, should be applied. Although a particle filter can provide better performance in general, it requires significant computational resources. On the other hand, the UKF is widely considered superior to the EKF [36,37] with possibly slightly higher computational cost. Furthermore, UKF effectively does both Jacobian and Hessian evaluations precisely through its sigma point propagation, without calculating the analytic Jacobian matrix as in EKF [38]. Therefore, we choose to employ UKF as the tracker; however, it should be noted that when the far-field assumption is not valid, the advanced filters should be considered because of the complex measurement models, although additional computational resources may be required.

The fusion center receives all the measurements available from the sensors in the network. Therefore, in the UKF, a stacked measurement vector, $Y_{k}^{s}$ for a scalar measurement network or $Y_{k}^{v}$ for a vector measurement network, should be used and can be defined as follows.

(11) $$\begin{array}{cc} Y_{k}^{s}= & {\left[∥B\left({\bar{x}}_{1,k}\right){∥}_{2},∥B\left({\bar{x}}_{2,k}\right){∥}_{2},\cdots ,{∥B\left({\bar{x}}_{n,k}\right)∥}_{2}\right]}^{T}+W_{k}^{s} \end{array}$$

(12) $$\begin{array}{cc} \mathrm{or},\;Y_{k}^{v}= & {\left[B{\left({\bar{x}}_{1,k}\right)}^{T},B{\left({\bar{x}}_{2,k}\right)}^{T},\cdots ,B{\left({\bar{x}}_{n,k}\right)}^{T}\right]}^{T}+W_{k}^{v} \end{array}$$

where $Y_{k}^{s}\in R^{n\times 1}$, $Y_{k}^{v}\in R^{3n\times 1}$,

$$W_{k}^{s}={\left[\omega_{1,k},\omega_{2,k},\cdots ,\omega_{n,k}\right]}^{T}∼N\left(0_{n},\sigma^{2}I_{n}\right)$$

and

$$W_{k}^{v}={\left[\omega_{1,k}^{T},\omega_{2,k}^{T},\cdots ,\omega_{n,k}^{T}\right]}^{T}∼N\left(0_{3n},\sigma^{2}I_{3n}\right).$$

The details of UKF are given as follows. For the 9-dimensional state $X_{k}$ defined in (1) with mean ${\hat{X}}_{k|k}$ and variance $\Sigma_{k|k}$ the unscented transform involves 2N+1, where N=9, and sigma points with weights

(13) $$\begin{array}{c} a_{i}=\left\{\begin{array}{c} \frac{\kappa }{N+\kappa }\;\;\;\;\;\;i=0\\ \frac{1}{2(N+\kappa )}\;\;\mathrm{otherwise} \end{array}\right. \end{array}$$

where $\kappa \in R^{+}$.

Suppose that the estimate of $X_{k}$ at time k−1 is ${\hat{X}}_{k-1|k-1}$ with covariance $\Sigma_{k-1|k-1}$, then at time *k*, the recursion is as follows.

Step 1: Calculate sigma points ${\hat{X}}_{k-1|k-1}^{j}$, j=0,⋯,2N by
  (14) $$\begin{array}{cc} {\hat{X}}_{k-1|k-1}^{j} & ={\hat{X}}_{k-1|k-1}+s\sqrt{N+\kappa }L_{i} \end{array}$$
  where s=0 for j=0, s=1 for j=1,⋯,N, s=−1 for j=N+1,⋯,2N and $L_{j}$ is the *j*-th column of L with $LU=\Sigma_{k-1|k-1}$ being the LU decomposition of covariance $\Sigma_{k-1|k-1}$.
Step 2: Predict the state and covariance by
  (15) $$\begin{array}{cc} {\hat{X}}_{k|k-1}= & \sum_{j=0}^{2N}a_{j}f\left({\hat{X}}_{k-1|k-1}\right) \end{array}$$
  (16) $$\begin{array}{cc} \Sigma_{k|k-1}= & \sum_{j=0}^{2N}a_{j}\left({\hat{X}}_{k-1|k-1}^{j}-{\hat{X}}_{k|k-1}\right){\left({\hat{X}}_{k-1|k-1}^{j}-{\hat{X}}_{k|k-1}\right)}^{T}+Q_{K}. \end{array}$$
Step 3: The measurement update. The sigma points of the location difference between the target location and the *i*-th sensor can be calculated by ${\hat{\bar{x}}}_{i,k-1|k-1}^{j}=H{\hat{X}}_{k-1|k-1}^{j}-s_{i}$. the measurement prediction is thus
  (17) $$\begin{array}{ccc} {\hat{Y}}_{k|k-1}^{s,j}= & {\left[{∥B\left({\hat{\bar{x}}}_{k-1|k-1}^{j}\right)∥}_{2},\cdots ,{∥B\left({\hat{\bar{x}}}_{k-1|k-1}^{j}\right)∥}_{2}\right]}^{T} & \mathrm{Scalar}\;\mathrm{model} \end{array}$$
  (18) $$\begin{array}{ccc} {\hat{Y}}_{k|k-1}^{v,j}= & {\left[B{\left({\hat{\bar{x}}}_{k-1|k-1}^{j}\right)}^{T},\cdots ,B{\left({\hat{\bar{x}}}_{k-1|k-1}^{j}\right)}^{T}\right]}^{T}. & \mathrm{Vector}\;\mathrm{model} \end{array}$$
  For simplicity, we use • to denote s and v. Then we have
  (19) $${\begin{array}{c} {\hat{Y}}^{•} \end{array}}_{k|k-1}=\sum_{j=0}^{2N}a_{j}{\hat{Y}}_{k|k-1}^{•,j}$$
  and
  (20) $$P^{•}=\sum_{j=0}^{2N}a_{j}{\left({\hat{Y}}_{k|k-1}^{•}-{\hat{Y}}_{k|k-1}^{•,j})({\hat{Y}}_{k|k-1}^{•}-{\hat{Y}}_{k|k-1}^{•,j}\right)}^{T}$$
  (21) $$T^{•}=\sum_{j=0}^{2N}a_{j}{\left({\hat{X}}_{k-1|k-1}^{j}-{\hat{X}}_{k|k-1})({\hat{Y}}_{k|k-1}^{•}-{\hat{Y}}_{k|k-1}^{•,j}\right)}^{T}.$$
**Step** **4:** Update the state estimation via
  (22) $$K=T^{•}{\left(P^{•}\right)}^{-1}$$
  (23) $${\hat{X}}_{k|k}={\hat{X}}_{k|k-1}+K(Y_{k}^{•}-{\hat{Y}}_{k|k-1}^{•})$$
  (24) $$\Sigma_{k|k}=\Sigma_{k|k-1}-KP^{•}K^{T}.$$

**Figure 5:**
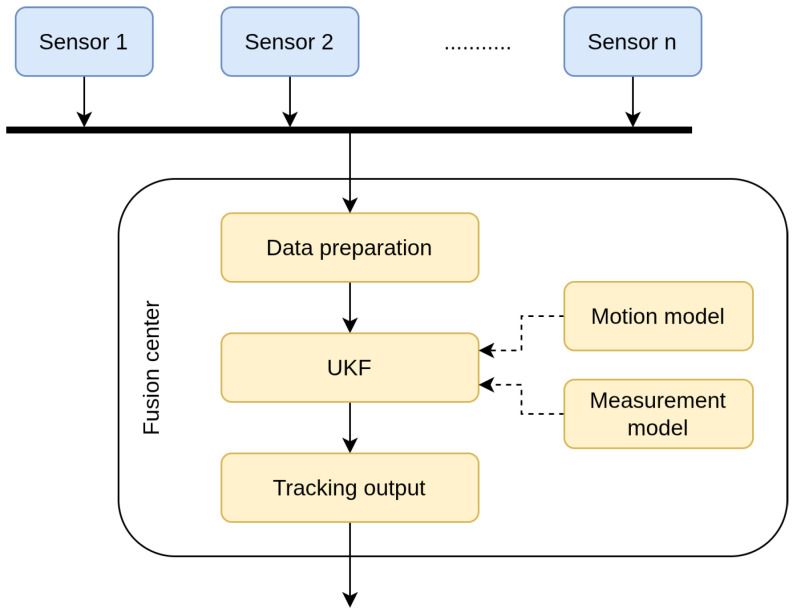
Magnetic object tracking via the UKF-based centralized fusion, where the arrows indicate the flow of signals.

In Figure 5, the flowchart of magnetic object tracking via the UKF-based centralized fusion is shown.

## 4. Fisher Information Matrix

To determine the best achievable sensitivity, we perform a Fisher Information Matrix (FIM) analysis [39].

Before introducing the FIM for scalar measurement and vector measurement, the Jacobian matrix of $B({\bar{x}}_{i,k})$ with respect to ${\bar{x}}_{i,k}$, where *i* is the sensor index and *k* is the time step, is given as follows:

(25) $$\begin{array}{c} J_{i,k}=\frac{3}{r_{i,k}^{7}}\left(r_{i,k}^{2}{\bar{x}}_{i,k}^{T}MI_{3}+r_{i,k}^{2}{\bar{x}}_{i,k}M^{T}+r_{i,k}^{2}M{\bar{x}}_{i,k}^{T}-5{\bar{x}}_{i,k}^{T}M{\bar{x}}_{i,k}{\bar{x}}_{i,k}^{T}\right). \end{array}$$

It can be shown that the rank of $J_{i,k}=3$ if ${\bar{x}}_{i,k}\neq {\left[0,0,0\right]}^{T}$.

- Scalar measurement:
  From (9) we have $y_{i,k}∼N(∥B\left({\bar{x}}_{i,k}\right){∥}_{2},\sigma^{2})$. Denote the scalar Fisher Information (FI) for the scalar measurement at time *k* by $I_{i,k}^{s}$
  (26) $$\begin{array}{c} I_{i,k}^{s}=\frac{1}{\sigma^{2}}\nabla ∥B\left({\bar{x}}_{i,k}\right){∥}_{2}^{T}\nabla {∥B\left({\bar{x}}_{i,k}\right)∥}_{2} \end{array}$$
  where the $\nabla ∥B\left({\bar{x}}_{i,k}\right){∥}_{2}$ is the gradient of $∥B\left({\bar{x}}_{i,k}\right){∥}_{2}$ with respect to ${\bar{x}}_{i,k}$ and given by
  (27) $$\begin{array}{c} \nabla ∥B\left({\bar{x}}_{i,k}\right){∥}_{2}=\frac{B{\left({\bar{x}}_{i,k}\right)}^{T}J_{i,k}}{∥B\left({\bar{x}}_{i,k}\right){∥}_{2}}. \end{array}$$
- Vector measurement:
  For the vector case, from (4), we can see that $y_{i,k}∼N\left(g\left({\bar{x}}_{i,k}\right),\Sigma \right)$. Denote the Fisher Information Matrix (FIM) for the vector measurement at time *k* by $I_{i,k}^{v}$. Then we have
  (28) $$\begin{array}{c} I_{i,k}^{v}=\frac{1}{\sigma^{2}}J_{i,k}^{T}J_{i,k}. \end{array}$$

Since measurements are obtained by each sensor independently, the total FIM, $I_{k}^{s}$ or $I_{k}^{v}$, for scalar or vector networks, is the summation of the FIMs of all sensors and can be written as

(29) $$\begin{array}{c} I_{k}^{s}=\sum_{i=1}^{n}I_{i,k}^{s}\;\;\;\mathrm{or}\;\;\;I_{k}^{v}=\sum_{i=1}^{n}I_{i,k}^{v}. \end{array}$$

Intuitively, one scalar sensor cannot provide sufficient information in estimating a unique location of the target, which can be seen from the FIM corresponding to the scalar measurement model. From [40], the FIM is non-singular if and only if the underlying parameters are (locally) observable. Therefore, $\nabla ∥B\left({\bar{x}}_{i,k}\right){∥}_{2}$ is a row vector and then $\mathrm{Rank}(\nabla ∥B\left({\bar{x}}_{i,k}\right){∥}_{2}^{T}\nabla ∥B\left({\bar{x}}_{i,k}\right){∥}_{2})=1$. On the other hand, $\mathrm{Rank}(I_{k}^{v})=3$ (since $J_{i,k}^{T}$ is full rank). As a result, the single sensor with vector measurement is sufficient to provide a unique estimate of the location of the target. This is one of the major advantages of vector magnetometers providing magnetic field measurements, and leads directly to the resilience and tracking sensitivity improvements that we find below.

The Cramér–Rao bound (CRLB) is the best achievable performance bound on the variance of all unbiased estimators for the underlying measurement model and is widely used for benchmarking. Mathematically, the CRLB is the inverse of the FIM. Since the FIM for the single scalar measurement is singular, then its CRLB will be infinity. However, an array of sensors, at least 3, with scalar measurement can be used to provide sufficient information and therefore the FIM will be full rank and the CRLB exists. This is shown in the following simulation results. For efficient interpretation and comparison, the squared root of the trace of the CRLB, $\sqrt{\mathrm{Tr}(CRLB)}$, is used as a measure of the total standard deviation (std) [41] in the following analysis.

**Figure 6:**
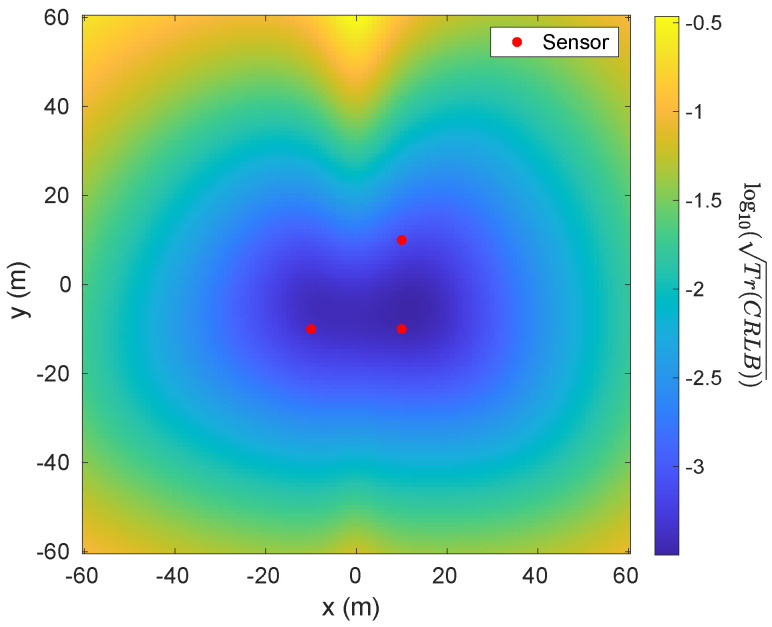
Calculated $\sqrt{\mathrm{Tr}(CRLB)}$ with three sensors (scalar measurement) located at [10,10,−25] m, [10,−10,−25] m and [−10,−10,−25] m as well as $M={\left[600,0,0\right]}^{T}$ Am. The results are plotted in $\log_{10}$ scale.

**Figure 7:**
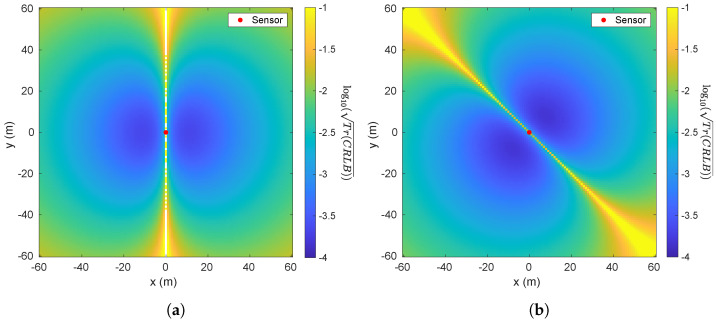
Calculated $\log_{10}\left(\sqrt{\mathrm{Tr}(CRLB)}\right)$ with 3 sensors (vector measurement) located at [0,0,−25] m and different *M*. (**a**) $M={\left[600,0,0\right]}^{T}$ Am; (**b**) $M={\left[600,600,2\right]}^{T}$ Am.

Figure 6 shows an example of the squared root of trace of CRLB, i.e., $\sqrt{\mathrm{Tr}(CRLB)}$, with 3 sensors (scalar measurement) located at [10,10,−24] m, [10,−10,−24] m and [−10,−10,−24] m, and $M={\left[600,0,0\right]}^{T}$ A·m^2^. We can see that 3 sensors are enough to provide sufficient information in estimating the target because of the non-singular FIM. On the other hand, Figure 7 shows two examples of trace of CRLB with 1 sensor (vector measurement) located at [0,0,−24] m and different *M*. It can be seen that, in Figure 7a, the trace of CRLB is symmetric about the *x*-axis while, in Figure 7b, it is symmetric about the y=x line roughly, which means that the value of *M* impacts the symmetricality of $\sqrt{\mathrm{Tr}(CRLB)}$.

Another notable phenomenon in Figure 7a is that the target is unobservable, labeled by white points, if it lies on the *x*-axis due to the FIM being singular since $M={\left[600,0,0\right]}^{T}$ Am. However, if *M* is non-zero vector and cannot be scaled to all-one vector, there will be no unobservable point.

## 5. Performance Comparison of Scalar and Vector Arrays

In this section, we consider two scenarios to demonstrate the relative performance of the networks with different measurement models, i.e., scalar and vector. In the first scenario, by changing the measurement model, the position of the dipole (*z*-axis), the CRLB of the two models are demonstrated and plotted to show that the vector measurement outperforms the scalar one. In the second scenario, a practical example is considered to show how the performance of the sensor array changes by changing the number and spacing of sensors as well as the target’s trajectories.

In the following simulations, since underwater/surface vehicles are slow-moving and less agile in maneuvering [42,43], the constant acceleration motion model is used in the simulations. Specifically, the Wiener acceleration model and process noise covariance Q are used in this paper [44], i.e.,

(30) $$\begin{array}{c} X_{t,k}=\left(\left[\begin{array}{ccc} 1 & T & \frac{1}{2}T^{2}\\ 0 & 1 & T\\ 0 & 0 & 1 \end{array}\right]⊗I_{3}\right)X_{t,k-1}+u_{k} \end{array}$$

and the variance of the process noise $u_{k}$ is

(31) $$\begin{array}{c} Q=\left[\begin{array}{ccc} \frac{1}{4}T^{4} & \frac{1}{2}T^{3} & \frac{1}{2}T^{2}\\ \frac{1}{2}T^{3} & T^{2} & T\\ \frac{1}{2}T^{2} & T & 1 \end{array}\right]⊗I_{3} \end{array}$$

where T=0.1 s is the sampling time, ⊗ is the Kronecker product and $I_{3}$ is the 3-dimensional identity matrix. It should be noted that the variance of the process noise Q is important in practice and may impact the filter performance; however, it is beyond the scope of this paper. Interested readers are referred to [45] for further details on filter tuning.

### 5.1. Scenario I

In this scenario, 49 sensors are placed in a square grid over area [−400,800]m×[400,−800]m. The target is assumed to be on the surface (i.e., z=0), with the sensor array depth either at z=−25 m or z=−80 m. In the following simulations of CRLB, the std of measurement noise is 10 pT. Since the CRLB is proportional to the std of measurement noise, similar results can be derived for other noise levels. The ground truth motion of the target is circular above the sensor array and plotted with a red dashed line.

**Figure 8:**
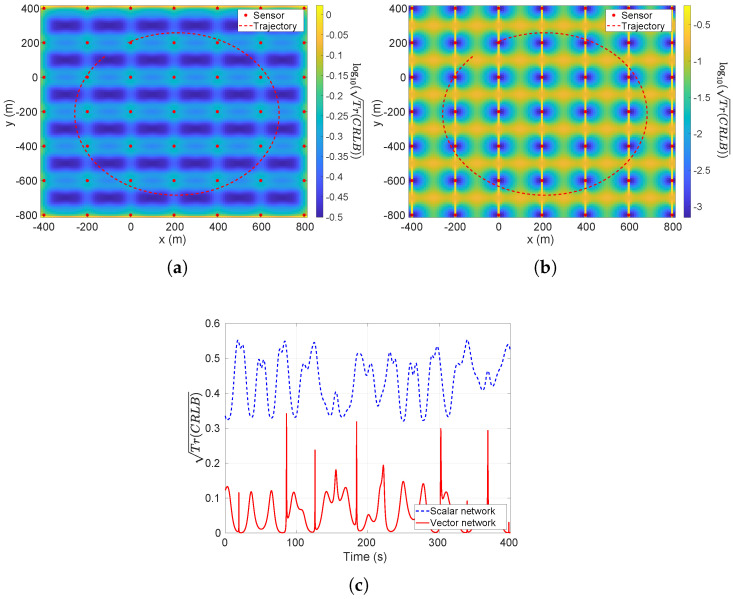
The plot of $\log_{10}\left(\sqrt{\mathrm{Tr}(CRLB)}\right)$ over the interested area and true trajectory. The std of noise is 10 pT and sensors’ depth is fixed at z=−25 m. (**a**) Scalar model; (**b**) vector model; (**c**) the $\sqrt{\mathrm{Tr}(CRLB)}$ along the trajectory.

In Figure 8a,b, the $\sqrt{\mathrm{Tr}(CRLB)}$ values of the interested area for scalar and vector measurement models are plotted respectively. In these two figures, the height of the sensors is fixed to be −25 m. In Figure 8c, the $\sqrt{\mathrm{Tr}(CRLB)}$ values along the trajectory are plotted for scalar and vector measurement models. It can be seen that the latter one outperforms the former one in terms of the CRLB.

**Figure 9:**
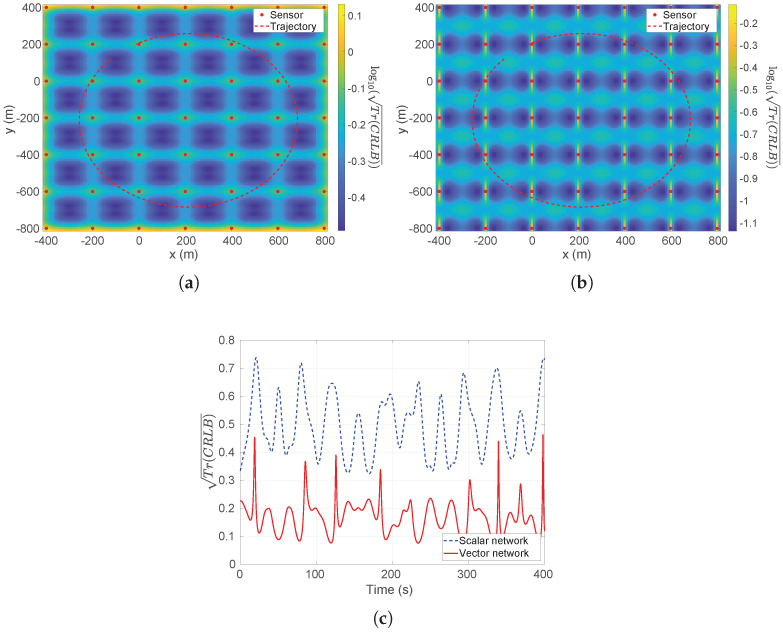
The plot of $\log_{10}\left(\sqrt{\mathrm{Tr}(CRLB)}\right)$ over the interested area and true trajectory. The std of noise is 10 pT and sensors’ heights are fixed to −80 m. (**a**) Scalar model; (**b**) vector model; (**c**) the $\sqrt{\mathrm{Tr}(CRLB)}$ along the trajectory.

Another example to see the improvement of the vector magnetometer array is for increasing depth. Figure 9a,b compare the scalar and vector arrays at a depth of z=−80 m, i.e., over three times deeper than the case in Figure 8, with the same noise level. Similarly, the $\sqrt{\mathrm{Tr}(CRLB)}$ values along the trajectory are plotted in Figure 9c for scalar and vector measurement models.

From these figures, it can be seen that the vector measurement model outperforms the scalar one in theoretical results, i.e., CRLB.

### 5.2. Scenario II

#### 5.2.1. Part I

**Figure 10:**
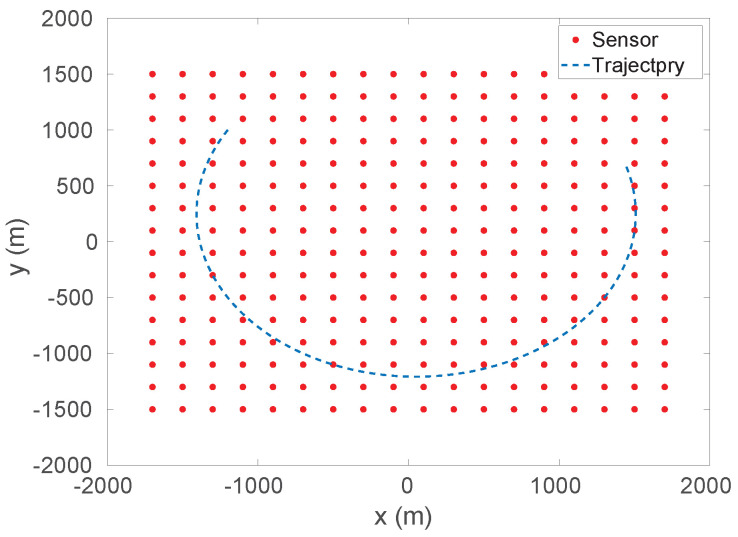
Diagram showing the sensor network superimposed to highlight one of our proposed use cases. We assume an m×n sensor array with *d* spacing along the *x*-axis and *y*-axis respectively covering an area of 3500×3000 m^2^. In this particular example shown in the figure, there are 288 sensors with spacing 200 m. The red dots are sensors while the blue curve is the trajectory of the object to be tracked.

In this scenario, we assume that the sensors are placed on a seabed and the target is moving circularly with a speed ~11 knots on the sea surface—see Figure 10 for illustration. We also assume in the following simulations that the depth of the sensors is 24 m, which is the approximate average depth of Port Phillip Bay seabed [46]. In order to investigate the impact of the density of the sensor array, the number of sensors will be varied according to the spacing distance, i.e., less spacing distance corresponds to more sensors. In particular, the sensor spacings are set to be 200 m, 300 m and 400 m in the simulations, corresponding to the number of sensors as 288, 132 and 72. In addition, the std of the measurement noise is set to be 32 pT, 158 pT and 320 pT, and the magnetic moment of the target is ${\left[600,0,0\right]}^{T}$ A·m^2^. Finally, the total running time of the trajectory is 16.6 min with 500 Monte-Carlo simulations.

**Figure 11:**
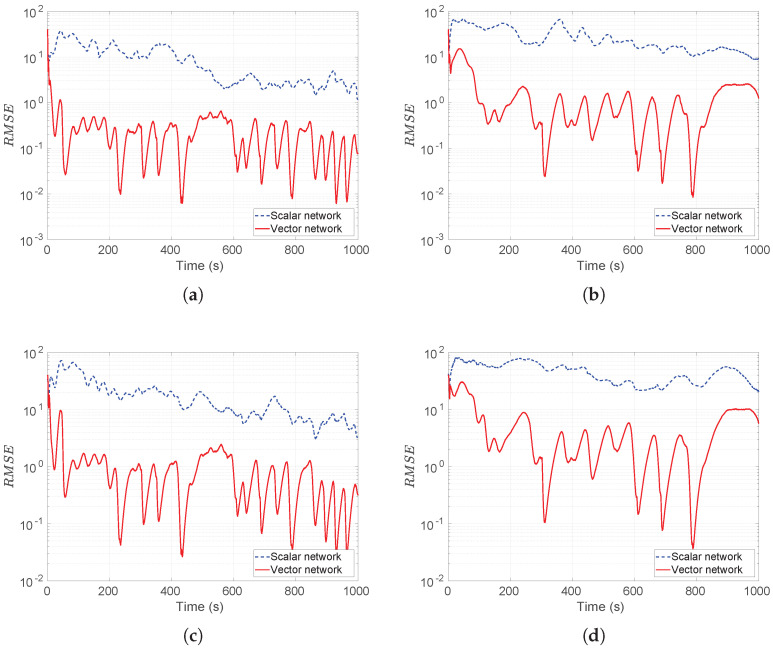
The simulated RMSEs with noise level 160 pT and different sensor spacings. (**a**) sensor spacing is 200 m with 32 pT; (**b**) Sensor spacing is 300 m with 32 pT; (**c**) sensor spacing is 200 m with 160 pT; (**d**) sensor spacing is 300 m with 160 pT. In all cases, vector magnetometer arrays significantly outperform scalar magnetometer arrays for target tracking.

The simulation results with std of noise 32 pT and 160 pT as well as sensor spacing 200 m and 300 m are shown in Figure 11.

The other cases are not plotted as failure percentages, percentages of divergence of tracking, or tracking error if they are greater than 200 m; the scalar vector is bigger so that the comparison is not meaningful—see Table 1.

From Figure 11 and Table 1, we can see that the vector network outperforms the scalar one in terms of accuracy and successful tracking performance. In the figures, one can notice that the minimal RMSE that the vector model can achieve is as low as 0.001 m, compared to the scalar model one being 0.3 m. It also can be seen that, in Figure 11a, the RMSE of the vector model performs at least (approximately) 3 times as many as the scalar model, at the 500 s point, while at most (approximately) 15 times at the 780 s point. Similar results can be observed in other figures.

**Figure 12:**
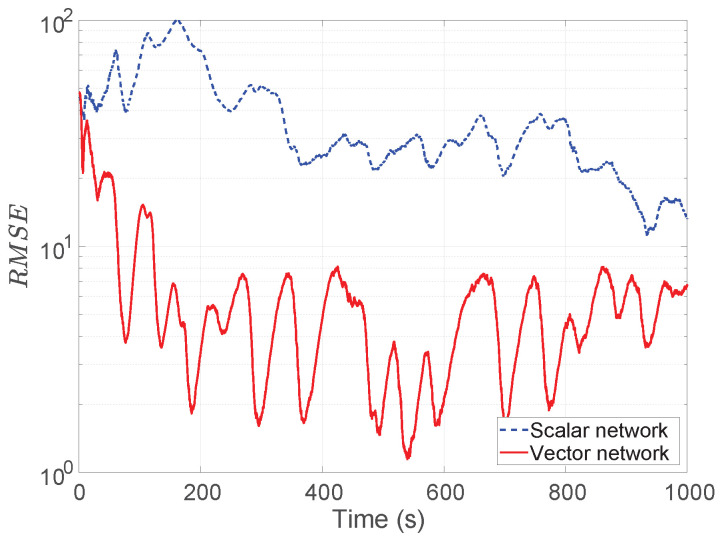
Simulated RMSEs with sensor depth −80 m. The horizontal sensor spacing is 200 m and the noise is 160 pT. RMSE is larger than the corresponding case shown in Figure 9, but similar trends are observed.

To illustrate the impact of varying sensor depth, a simulation result with sensors at −80 m is show in Figure 12, where the horizontal spacing is 200 m and the noise is 160 pT.

Comparing this with the case of −24 m depth, it can be seen that the tracking errors for both of the sensor models are larger when the depth is −80 m, while the vector sensor networks outperform the scalar one, which agrees with the CRLB results shown in Figure 9.

#### 5.2.2. Part II

The undersea domain can be harsh, and replacing sensors may be difficult. Therefore it is important to consider the consequences of permanent sensor outages, to determine if the sensor arrays are able to fail gracefully.

In this scenario, we therefore consider the initial grid networks, and fail a randomly chosen subset of the sensors. By providing the RMSE tracking fidelity over the full trajectory and Monte-Carlo averaging over the failed detectors, we are able to get a statistical measure for the resilience of the network.

Our results show significantly improved resilience for the vector network over the scalar network, highlighting the benefit of scalar magnetometer networks for resilient monitoring networks. In the simulations, three cases are considered, i.e., 10 sensors failed (Case I); 15 sensors failed (Case II); and 20 sensors failed (Case III). The IDs of the sensors to be failed are uniformly drawn from sets {1,2,3,⋯,n} without replacement, where *n* is the number of sensors. Table 2 shows the percentage of tracking failures as a function of increasing failed sensors. As can be seen, at these sensitivities, the vector network maintains tracking even with 20 failed sensors out of the 288 in the original grid. With this limit, the scalar network fails to track in 25.9 % of cases. This highlights the improved robustness of the vector network relative to scalar magnetometer networks.

## 6. Conclusions

The monitoring of commercial and non-commercial sea traffic is becoming increasingly important for maritime safety. Magnetometer arrays have the potential to provide crucial information for monitoring traffic and detecting threats. Our analysis highlights the added advantage of vector magnetometers over scalar magnetometers.

Vector arrays typically provide more than a three-fold improvement in performance due to their enhanced ability to localise targets and are more resilient to the loss of sensors than comparable scalar arrays. This is a strong motivating factor for exploring practical vector magnetometer solutions suitably for the undersea domain, such as diamond in fibre or other ruggedised diamond-based solutions for magnetometry.

Although tracking accuracy has been greatly improved in three-axis sensor networks, the deployment of vector magnetometers in the undersea domain is challenging, as it is affected by hardware robustness due to the high pressure, salinity, difficulties in exact positioning and alignment, undersea currents, and marine encrustation. Several efforts have been made to address these challenges, such as [47,48,49,50,51]. We will continue our research in this direction.

## Figures and Tables

**Table 1:** The failure percentages of the simulations with different noise levels and sensor spacings. Here we see that vector networks provide significantly enhanced tracking resilience.

Noise Level	Sensor Spacing	Scalar Network	Vector Network
32 pT	200	8.3%	0%
	300	19.7%	0%
	400	26.0%	0.3%
160 pT	200	21.0%	0%
	300	51.0%	0%
	400	84.7%	0.3%
320 pT	200	35.6%	0.5%
	300	76.7%	8%
	400	93.7%	12.2%

**Table 2:** The failure percentages of the cases.

	Noise Level	Scalar Network	Vector Network
Case I	32 pT	9.3%	0%
	160 pT	23.5%	0%
Case II	32 pT	10.1%	0%
	160 pT	24.1%	0%
Case II	32 pT	12.4%	0%
	160 pT	25.9%	0%

## Data Availability

The datasets presented in this article are not readily available because the data are part of an ongoing study. Requests to access the datasets should be directed to ADG.

## Abbreviations

The following abbreviations are used in this manuscript:

CRLB	Cramér-Rao lower bound
EKF	Extended Kalman filter
FIM	Fisher Information Matrix
RMSE	Room mean square error
SWaP	Size weight and power
UKF	Unscented Kalman filter
